# Personalized Cancer Therapy for Urological Cancers: From Bench to Bedside and Back

**DOI:** 10.1155/2012/298105

**Published:** 2012-09-12

**Authors:** Hirotsugu Uemura, Colleen Nelson, Jack A. Schalken, Laurence Klotz

**Affiliations:** ^1^Department of Urology, Kinki University Faculty of Medicine, 377-2 Ohno-Higashi, Osaka-Sayama, Osaka 589-8511, Japan; ^2^Australian Prostate Cancer Research Centre-Queensland, Queensland University of Technology and Princess Alexandra Hospital, Level 1, Building 1, Ipswich Road, Brisbane, QLD 4102, Australia; ^3^Department of Experimental Urology, The Radboud University Nijmegen Medical Centre, P.O. Box 9101, 6500 HB Nijmegen, The Netherlands; ^4^Division of Urology, Sunnybrook Health Sciences Centre, 2075 Bayview Avenue MG 408, Toronto, ON, Canada M4N3M5

A better understanding of key regulatory pathways perturbed in cancers has led to the development of a molecularly targeted therapeutic approach. As a result, new generations of compounds aimed at inhibiting specific pathways that play prominent roles in urological cancers have been developed and continue to evolve. The possibility of being able to perform molecular profiling in tumors would, at least in theory, provide the clinician with information needed to manage a personalized therapeutic regimen according to an individual patient's needs. While this modality is already being implemented in other cancer fields, personalized medicine for patients suffering from advanced lower urogenital tumors is still in the developmental phase. This impetus has prompted a concerted effort from investigators in the fields of basic, translational, and clinical research to develop and implement a tailored treatment approach. In this issue, we present a collection of seven articles that contribute to our knowledge of personalized medicine for urological cancers.

Androgen deprivation therapy (ADT) for advanced prostate cancer is one of the earliest forms of targeted therapy and has remained the choice of treatment by physicians. Unfortunately, most patients will eventually become non-responsive to ADT and succumb to the disease. Since its inception, knowledge for the understanding of androgen receptor (AR) signaling and mechanisms driving the resistance to ADT has been significantly improved. As a result, a new generation of therapeutic agents has been developed. The paper by X. Hou and T. W. Flaig gives a historical account of ADT for metastatic prostate cancer and summarizes current advancements in hormonal therapies using newly developed agents such as MDV3100, TOK-001, and TAK-700. 

The association between insulin and androgens in prostate cancer progression is detailed by J. H. Gunter et al. In this review, the authors describe the inverse relationship between insulin and testosterone levels and the metabolic crosstalk between the two signaling axes. The authors discuss the effects of ADT-induced hyperinsulinaemia and describe the direct effects of insulin on prostate tumor cells and its clinical implications. 

The 5 *α*-reductase inhibitors represent an important family of enzymes that mediate the conversion of testosterone to the more potent dihydrotestosterone (DHT). Consequently, several compounds have been developed to inhibit the activity of the enzymes to prevent and treat a number of diseases. The paper by F. Azzouni et al. goes into great detail to describe the basic biochemical properties and functions of the 5 *α*-reductase isozyme family and its clinical significance with regards to prostate cancer prevention and treatment.

Gene inactivation of PTEN is a common occurrence in prostate cancers and is associated to metastatic potential, androgen independence, and poor prognosis. The review by M. A. De Velasco and H. Uemura chronicles the evolution of the PTEN-deficient genetically engineered mouse (GEM) and the cooperation between PTEN and other genetic alterations that contribute to tumor progression. The authors also describe the usefulness of GEM models for biomarker and drug discovery. 

Also in this issue, two reviews delve into the progress towards personalized medicine for muscle invasive and metastatic bladder cancer. J. S. Chang et al. review potential molecular biomarkers currently under investigation and discuss their future. In the second paper A. Kawashima et al. review the excision repair cross-complementing group 1 (ERCC1) gene and its role in tumor development and therapeutic resistance to cytotoxic DNA-damaging chemotherapy and ionizing radiation. 

Finally, in the original paper by R. Nagarajan et al. the authors use the apparent diffusion coefficient, derived from diffusion-weighted (DWI) imaging, to identify higher-grade prostate cancer lesions. Although the data is preliminary, the authors' findings reveal a potential for the use of DWI imaging to differentiate those patients with high-grade lesions. 

